# Application of UV Laser for Ohmic Contact Formation on 4H-SiC

**DOI:** 10.3390/ma18214946

**Published:** 2025-10-29

**Authors:** Andrzej Kubiak, Janusz Wozny, Izabela Bobowska, Alessandro Verdolotti

**Affiliations:** 1 Department of Semiconductor and Optoelectronic Devices, Lodz University of Technology, 116 Zeromskiego, 90-924 Lodz, Poland; janusz.wozny@p.lodz.pl (J.W.); alessandro.verdolotti@dokt.p.lodz.pl (A.V.); 2 Department of Molecular Physics, Lodz University of Technology, 116 Zeromskiego, 90-924 Lodz, Poland; izabela.bobowska@p.lodz.pl

**Keywords:** silicon carbide, ohmic contact, UV laser

## Abstract

In this paper, we demonstrate a simplified method for fabricating ohmic contacts on 4H-SiC substrates using pulsed UV laser surface modification followed by application of a silver-based conductive adhesive. Even a small number of laser passes significantly improved the contact interface, while ten or more repetitions produced linear I–V characteristics with low voltage drops. SEM analysis revealed surface ablation and an expanded effective area of the contact. Raman spectroscopy proved that laser processing leads to surface amorphization of the SiC sample. DFT simulations showed that the amorphous SiC layer is a material with no band gap, explaining the elimination of the Schottky barrier. Our approach enables the manufacturing of reliable, low-resistive contacts without high-temperature annealing and offers a practical route for rapid SiC device prototyping.

## 1. Introduction

Silicon carbide (SiC) is a wide-bandgap semiconductor material that has found extensive application in power electronics. Its advantageous physical properties make it particularly suitable for devices operating under high power, high-frequency, and elevated temperature conditions. The current level of technological maturity of SiC enables commercial availability of selected semiconductor devices fabricated from this material. Nevertheless, several research challenges remain, particularly in the areas of large-area defect-free monocrystalline SiC substrate fabrication, reliable electrical contact formation, and understanding the influence of crystal defects on device reliability [1,2]. Despite these challenges, the favorable intrinsic characteristics of SiC and the availability of large single-crystal substrates continue to stimulate both fundamental research and industrial implementation efforts.

In metal–semiconductor contacts, the carrier transport is governed by the formation of a Schottky barrier, which comes from the difference between the metal work function and the semiconductor electron affinity. When the Fermi levels align, a depletion region forms in the semiconductor, creating a potential barrier that limits carrier transport.

For 4H-SiC, the wide bandgap (≈3.2 eV) and relatively low electron affinity result in a Schottky barrier, making ohmic conduction difficult to achieve without interface modification. Conventional approaches rely on high-temperature annealing of metal layers such as Ni or Ti/Al/Ni to form interfacial silicides that locally modify the barrier width and enable electron tunneling, which is the main mechanism of charge transport at the metal–4H-SiC interface, leading to linear current–voltage characteristics.

The development of low-resistance ohmic contacts to 4H-SiC remains a central challenge in wide-bandgap semiconductor device engineering. Traditionally, ohmic characteristics have been achieved by depositing metal stacks (e.g., Ni and Ti/Al/Ni) followed by high-temperature annealing (typically ≥950 °C), which promotes the formation of interfacial silicides (e.g., NiSi) or carbon-related vacancies to lower the Schottky barrier and reduce specific contact resistance (ρ_n_) to the 10^−6^–10^−4^ Ω·cm^2^ range [3]. However, such thermal steps can degrade dielectric layers, cause dopant redistribution, and necessitate complex lithography [4].

To mitigate these limitations, the application of laser techniques for forming ohmic contacts on SiC has been investigated for several years. Literature reports indicate that laser-induced processes may offer many advantages over conventional rapid thermal annealing (RTA) methods, such as the possibility to repair lattice defects in SiC crystal [5], precise energy distribution allowing the occurrence of desired chemical reactions without heating the whole wafer [6], or the opportunity to process the metal–semiconductor interface from the backside of the sample [7]. With the rapid progress in laser technology and increasing accessibility of high-performance laser sources, research on their application in silicon carbide processing, particularly in the formation of electrical contacts, has gained significant momentum [8,9,10,11].

Laser-based processes have attracted significant attention as alternative contact formation methods. Pulsed laser annealing of metallic films on 4H-SiC (e.g., Ni, Ti, and Ti/Si/C) has achieved low ρ_n_ (down to ~2.4 × 10^−4^ Ω·cm^2^) through non-equilibrium formation of silicide or carbide phases [12,13]. For instance, an excimer UV laser (308 nm) enabled rapid silicidation of Ni films and facilitated ohmic behavior comparable to conventional rapid thermal annealing (RTA) [14]. Similarly, Ti-based contacts subjected to pulsed laser annealing showed specific resistivity improvements and contact morphology optimization depending on laser fluence and scan overlap [15].

Beyond metal silicide formation, other studies have revealed that annealing carbon layers on SiC can induce amorphous or graphitic phases that transform Schottky contacts into ohmic ones, with ρ_n_ in the 10^−3^–10^−4^ Ω·cm^2^ range [16]. Moreover, in thermally activated Ti/Al/Au contacts on p-type 4H-SiC, the emergence of an amorphous Si–C phase at the interface correlated with reduced contact resistance [17].

Despite these advances, prior research predominantly concentrates on laser treatment of pre-deposited metal or carbon layers—not on direct laser modification of the SiC surface itself, followed by application of thick-film conductive adhesives. Reports of thick-film (screen-printed) contacts formed directly onto laser-amorphized SiC surfaces remain scarce. Only recently have preliminary works suggested the feasibility of such an approach using silver-based adhesives on laser-treated 4H-SiC [18,19,20].

A frequent requirement in laboratory practice is the fabrication of small batches of semiconductor test structures of varying geometries and dimensions. For electrical characterization of such prototypes, the presence of reliable ohmic contacts is essential. In the classical technological approach, this requires a sequence of steps, including the deposition of metallic layers, mask-based photolithography, selective etching, and annealing [2,3]. Implementing these processes for single or low-volume samples is time-consuming and technologically demanding. Motivated by the need for process simplification, we investigated whether ohmic contacts could be realized on SiC substrates through laser-induced surface modification combined with the application of conductive adhesive. The presented technique by laser-induced surface amorphization modifies the SiC directly without metal diffusion. We believe that rapid melting and quenching destroy the long-range crystal order and produce an amorphous SiC layer that exhibits a high density of localized states and a nearly metallic-like density of states. This disordered region facilitates carrier transport across the interface and effectively eliminates the Schottky barrier. Compared with traditional metal–annealing methods, the UV-laser approach simplifies processing by avoiding metal deposition steps, allows localized modification without heating the entire substrate, and is well-suited for rapid prototyping and can be used in low-volume device fabrication.

In this context, exploring direct UV-laser-induced amorphization of 4H-SiC to eliminate the Schottky barrier—without relying on metal deposition or high-temperature annealing—and subsequently forming thick conductive adhesive contacts represents a novel and promising direction. This method could significantly simplify prototyping workflows by removing complex lithographic steps and thermal budgets, while still achieving linear I–V behavior and low contact resistance.

In this study, we present a novel approach for the fabrication of non-rectifying contacts on SiC substrates, eliminating the need for conventional processes such as metal deposition, photolithography, etching, and high-temperature annealing. The experimental results demonstrate that laser-assisted surface modification enables the fabrication of low-resistive ohmic contacts on 4H-SiC substrates.

## 2. Materials and Methods

The study was performed using polished, 350 μm thick n-type monocrystalline 4H-SiC substrates from SiCrystal GmbH, Nürnberg, Germany, with a lateral dimension of approximately 6 × 6 mm. The substrates had a nitrogen doping concentration N_d_ of approximately 6 × 10^18^ cm^−3^. Prior to laser processing, all substrates underwent a cleaning procedure consisting of organic solvent rinsing, deionized water wash, and nitrogen drying. Five test structure types were prepared. Four samples (denoted S03, S10, S30, and SK1) were subjected to laser modification of the surface, while one (denoted REF) remained unprocessed and served as a reference sample.

A pulsed ultraviolet laser (Shenzhen Gainlaser Laser Technology Co., Ltd., Shenzhen, China) with a wavelength of λ = 355 nm, a maximum average output power of 3 W over the period, and a pulse duration of 10 ns was employed for surface modification of the contact areas. The laser operated at a repetition frequency of 20 kHz, with the beam focused to a spot diameter of approximately 20 µm, resulting in an energy fluence of 47.8 Jcm^−2^ delivered per pulse. At the laser interaction zone, the beam profile of this laser had a Gaussian characteristic. A galvo scanner system (DK Lasertechnik, Krakow, Poland, software version 1.1.5.0) was used to guide the laser over the surface, enabling precise control of the irradiation trajectory and allowing for the definition of circular contact regions as well as repeated scanning over the same area. The scanning velocity of the laser beam over the sample surface was fixed at 100 mm/s.

**Figure 1:**
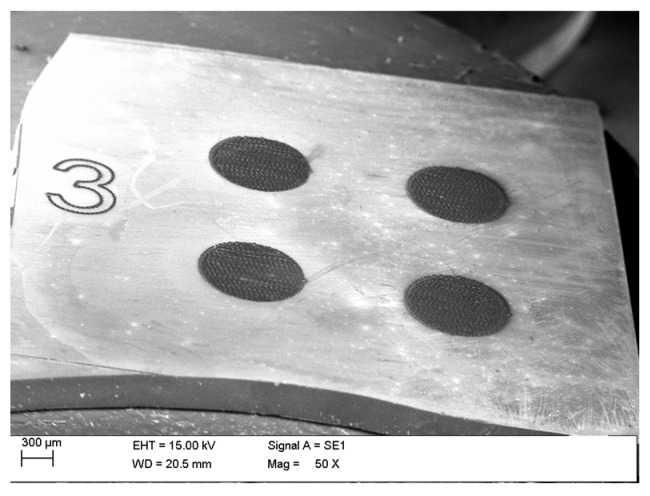
Example of the SiC substrate (sample S03) after local laser treatment.

On each of the substrates, four circular contact regions with a diameter of 1 mm were defined by laser irradiation, as presented in Figure 1. The separation between adjacent contact edges was maintained at 1 mm. To investigate the effect of repeated laser exposure, the number of irradiation passes was varied across the samples: 3 passes for substrate S03, 10 passes for substrate S10, 30 passes for substrate S30, and 100 passes for substrate SK1. In all cases, the laser operated at full output power (3 W). The reference substrate (REF) was not irradiated.

Following laser modification, electrical contacts were fabricated using a screen-printing technique. A mask made of 0.08 mm thick Kapton adhesive tape was prepared, in which circular openings corresponding to the contact areas were cut by laser machining. Through this mask, a silver-based conductive adhesive (Mechanic MCN-DJ002, containing 80–90% Ag with a particle size of 1–10 µm) was applied onto the designated fields. The same adhesive was also used to attach short sections of copper wire to each contact, enabling straightforward connection to the measurement equipment.

The overview of prepared samples and the corresponding number of laser beam repetitions applied to each substrate is presented in Table 1.

## 3. Results and Discussion

### 3.1. Surface Morphology

**Figure 2:**
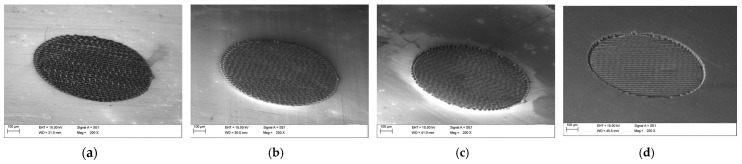
SEM images of contact fields before applying conductive adhesive for samples: (**a**) S03, (**b**) S10, (**c**) S30, and (**d**) SK1.

The morphology of the SiC substrates after laser processing was examined using a EVO MA10 (Carl Zeiss Microscopy Ltd., Cambridge, United Kingdom) scanning electron microscope (SEM). Figure 2 presents the images of areas of the circular contact fields prepared on structures Sxx prior to the application of conductive adhesive. The darker regions correspond to the laser-modified SiC surface.

It was observed that the increasing number of laser scan repetitions led to a progressive ablation of the silicon carbide surface, resulting in thicker modified contact regions. Furthermore, due to the repeated passes of the laser beam along a linear scanning path, characteristic depressions in the form of parallel lines were formed. A pronounced depression of the outer edges of the contact fields was also visible in all irradiated areas.

**Figure 3:**
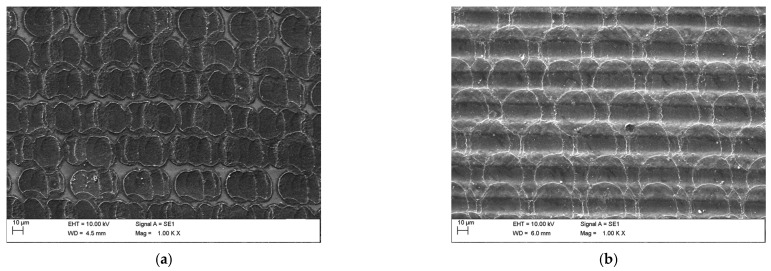
SEM images of the silicon carbide surface observed after scanning laser beam processing on samples (**a**) S03 and (**b**) SK1.

To evaluate the spatial influence of individual laser pulses, high-magnification (1000×) EVO MA10 SEM imaging was performed. Representative results for samples S03 and SK1 are shown in Figure 3. The interaction of each pulse induced localized melting of the silicon carbide surface within a diameter of approximately 30–40 µm. A defined contact area has been created by the laser beam following a predefined scanning technique along neighboring lines, which caused the formation of parallel, striped surface features on the processed surface. Those features are well-reproducible across samples and do not affect the uniformity of large-area contacts.

In the case of three laser passes (sample S03), fragments of the unmodified SiC surface remained within the contact area. In contrast, for samples subjected to higher repetition counts (S10, S30, and SK1), the entire surface area of the contact fields was modified, and deeper depressions arranged in parallel patterns were observed.

**Figure 4:**
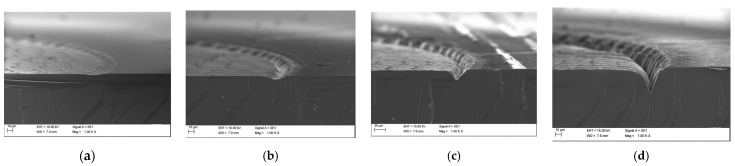
SEM images of cross-sections of contact area before applying conductive adhesive: (**a**) S03, (**b**) S10, (**c**) S30, and (**d**) SK1.

To assess the penetration depth of the laser–SiC interaction, cross-sectional analyses were performed on contact fields prepared with 3, 10, 30, and 100 laser passes (Figure 4). Cross-sections were obtained by introducing a single-line laser cut on the substrate side opposite to the processed area, followed by mechanical cleaving along the cut. This approach enabled the preparation of sections without additional damage caused by invasive laser-cutting techniques.

The SEM images in Figure 4 show not only surface ablation, but also deeper ring-like formations along the edges of the prepared contact area. This feature is caused by additional operation of the laser beam along the line that defines each contact area, but it did not influence fabricated contacts, as the conductive adhesive was applied on the flat part of the laser-modified area. Despite these observations, no distinct microstructural changes in the SiC bulk material could be identified. Based on the low laser power, short pulse duration, and strong UV absorption in SiC, it can be assumed that the effective modification depth was on the order of several nanometers.

Following SEM observations, conductive adhesive was applied to the contact fields as described in the methodology. Each sample was equipped with four contacts, enabling electrical characterization using a Van der Pauw measurement setup. Short sections of copper wire were attached to the contacts using the same silver-based conductive adhesive. The conductive adhesive has been dried for 24 h at room temperature.

**Figure 5:**
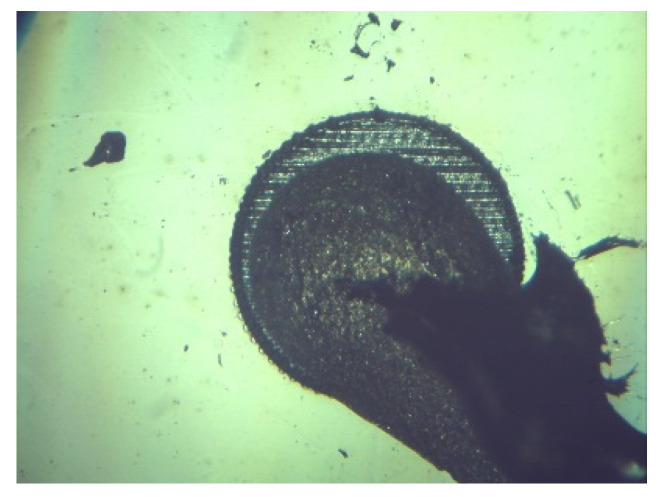
Optical microscopy image of the laser-modified SiC surface after conductive adhesive application and drying.

An optical microscopy (DeltaOptical, Warsaw, Poland) image of a representative contact structure on the SiC surface is shown in Figure 5. The observation was carried out after the drying process of the silver-based conductive adhesive was completed. The image confirms uniform adhesive distribution within the laser-modified contact area, ensuring reliable bonding between the semiconductor surface and external measurement leads.

### 3.2. I–V Characterization

The current–voltage (I–V) characteristics of the fabricated contacts were measured for all samples, including the unmodified reference (sample REF) and those subjected to 3, 10, 30, and 100 laser beam repetitions (samples Sxx). The Keithley 2450 source-meter unit (Keithley Instruments, Cleveland, OH 44139, USA), combined with the MPI TS150 (MPI Corp., Chupei, Taiwan) manual probing system, was used at this step. The measurements were performed at a speed of 1 NPLC, which is the default setting for this SMU. The SMU operated in current source mode, which, based on our experience, provides better control over current in the low-current range. The current was swept from −I_max_ to +I_max_ for both forward and reverse contact configurations (e.g., 1–2 and 2–1), which helped to reduce systematic error.

The lowest current was observed in the sample that was not treated with the laser. For this sample, the measured currents were in the maximum range of I_max_ = 50 nA, requiring voltages of up to ±6 V. Since the front connections were used, the SMU operated in the 1 µA range, resulting in an accuracy of approximately 400 pA. This corresponds to a 4% error at the lowest current used, 10 nA. Higher forced currents are set with better accuracy.

Voltage measurements performed by the SMU were most demanding for the best-conducting sample, where the current was in the range of I_max_ = 50 mA (accuracy ± 15 µA), and the measured voltages were up to 2.5 mV, with an estimated accuracy of 0.17 mV.

**Figure 6:**
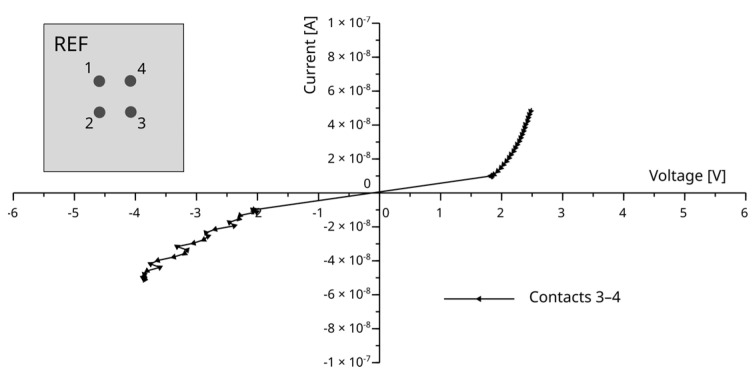
I–V characteristic of contacts prepared on reference sample (REF), which was not modified using UV laser processing. Schematic SiC sample with localization of contacts 1-2-3-4 is presented.

The results obtained for the reference sample (REF) are presented in Figure 6. Characterization indicated that when the conductive adhesive was applied directly onto the unmodified SiC surface, the I–V characteristics exhibited a strongly nonlinear response, which is typical of a rectifying contact. Owing to the compliance limits of the source-measure unit, the data for this sample are presented only for a single contact pair.

**Figure 7:**
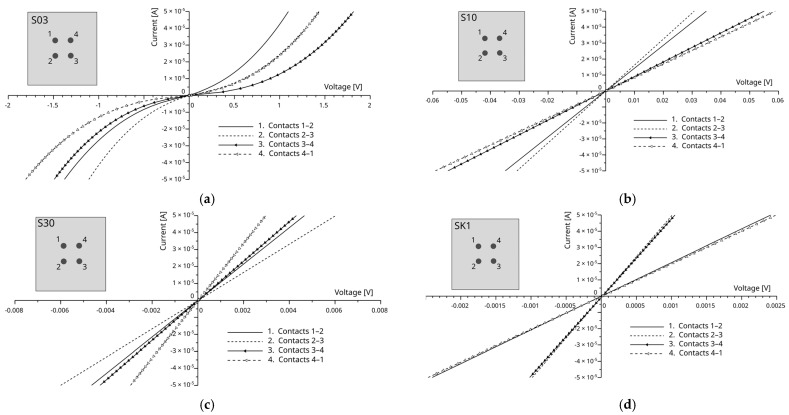
I–V characteristics of contacts on samples: (**a**) S03, (**b**) S10, (**c**) S30, and (**d**) SK1. Schematic SiC samples with localization of contacts 1-2-3-4 are presented.

The results of the electrical characterization of samples whose contact areas have been laser-processed are presented in Figure 7. Even a limited number of laser passes significantly altered the electrical properties of the contact interface.

For sample S03, the current at a given bias increased by nearly three orders of magnitude compared to the unmodified reference, as shown in Figure 7a. This result indicates that surface modification, even at shallow depths, substantially reduces the effective barrier at the adhesive–SiC interface. A fully linear ohmic characteristic was obtained for sample S10. At a forced current of 0.050 mA, the voltage drop decreased from approximately 2 V (REF sample) to 60 mV (sample S10). Further increases in the number of laser passes led to additional reductions in contact resistance: for 30 passes (sample S30), the voltage drop was reduced to approximately 4 mV, while for 100 passes (sample SK1), it reached as low as 1.5 mV.

These results suggest that increasing the number of laser repetitions progressively modifies the SiC surface to greater depths, thereby enlarging the effective conductive interface area and enhancing carrier transport across the contact. The observed evolution of the I–V response from rectifying to nearly ideal ohmic demonstrates the effectiveness of the laser-assisted surface modification approach.

### 3.3. Raman Spectroscopy

The Raman spectroscopy was used to reveal the crystalline structure of the laser-processed samples. For the measurement, the Jobin Yvon T64000 spectrometer (HORIBA Ltd., Kyoto, Japan) equipped with an Olympus B-40 (×50, NA = 0.5) microscope (Olympus Corporation, Tokyo, Japan) was used, combined with an Innova 90C argon laser (514.5 nm) (Coherent Inc., Santa Clara, CA 95054, USA). Two samples were characterized: the reference sample (REF) and the laser-modified sample SK1. Acquisition time was set to 3 × 20 s, and laser power measured on the sample surface was 3 mW.

**Figure 8:**
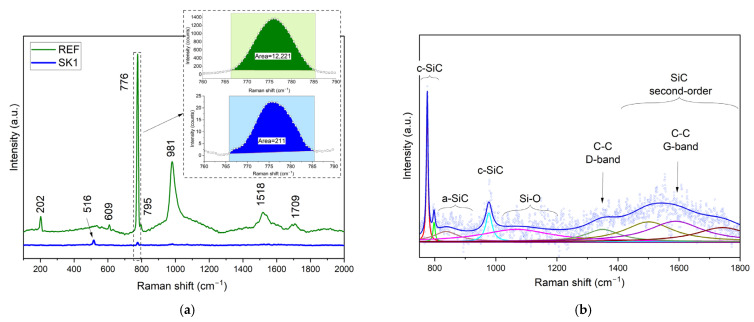
(**a**) Raman spectra obtained from REF sample (green) and laser-modified SK1 sample (blue). Inset: the area values for selected Raman bands. (**b**) Fitting of SK1 Raman spectrum (blue line—fitted curve; blue circles—original data; colored lines—individual fitting lines).

The green line in Figure 8a presents the Raman spectra of the untreated, non-irradiated surface of the 4H-SiC sample (REF). Observed bands located at 202 cm^−1^, 609 cm^−1^, 776 cm^−1^, 795 cm^−1^, and 981 cm^−1^ are typical for 4H-SiC structure and are confirmed by previous studies [21,22]. The several weaker peaks located in the region 1400–1800 cm^−1^ correspond to second-order Raman bands [23].

The Raman spectrum obtained for the surface modified with laser on sample SK1 (blue line in Figure 8) shows a significant reduction in band intensity when maintaining the same Raman measurement conditions. A more detailed quantitative analysis indicates that the peak area of the most characteristic band for 4H-SiC, located near 776 cm^−1^, decreased by a factor of 60 due to laser treatment. This can be attributed to surface amorphization and SiC decomposition that is commonly observed for laser-treated SiC surfaces [22,24]. Irradiation conditions play a crucial role in the final phase composition in the beam impact area. In our experimental conditions and based on Raman analysis, a new band was revealed: 516 cm^−1^. It can be assigned to nanocrystalline silicone. Bulk silicone has a characteristic phonon mode located at 521 cm^−1^, but for finite-size crystalline grains, this peak is typically shifted to lower wavenumbers [25].

The presence of a silicone peak can be explained by the fact that at temperatures above ca. 2730 K, the SiC crystal decomposes, leaving Si atoms in the remaining solid, and with carbon atoms evaporating as CO_2_ gas. The remaining bands visible on the spectrum of sample SK1 (Figure 8—blue line), although very weak, are in the same positions as for untreated 4H-SiC and can be assigned to the crystalline SiC phase existing under the thin, laser-modified layer of SiC sample.

Figure 8b shows the enlarged Raman spectrum of SK1 and the results of fitting: the blue circles represent the original data, and the fitted curve is the blue line. In addition to the signal from crystalline silicon carbide (c-SiC), bands attributed to amorphous SiC (a-SiC), silicon oxides (Si-O), and D and G bands representing glassy graphite can be observed. Disordered amorphous structures produce weak and broad Raman signals, so the weakly resolved Raman spectrum of the laser-irradiated SiC sample indicates the amorphous nature of the material.

### 3.4. Atomic Simulations

Understanding of the observed contact behavior requires verification of the potential barrier that forms between the conductive adhesive and the laser-modified SiC substrate. To investigate this phenomenon, atomistic simulations were carried out using the QuantumATK package [26]. To perform DFT calculations and to obtain the density of states (DOS), we used a linear combination of atomic orbitals (LCAO) basis set and employed the Heyd–Scuseria–Ernzerhof (HSE) hybrid exchange–correlation functional [27]. Localized atomic orbitals are efficient for large systems, while the HSE hybrid functional provides improved accuracy for calculating band gaps and localized electronic states. Brillouin-zone sampling was performed using a 2 × 2 × 1 Monkhorst–Pack k-point mesh, and the real-space integration grid was defined by a density mesh cutoff of 80 Hartree. For the analyzed structure, periodic boundary conditions were applied.

**Figure 9:**
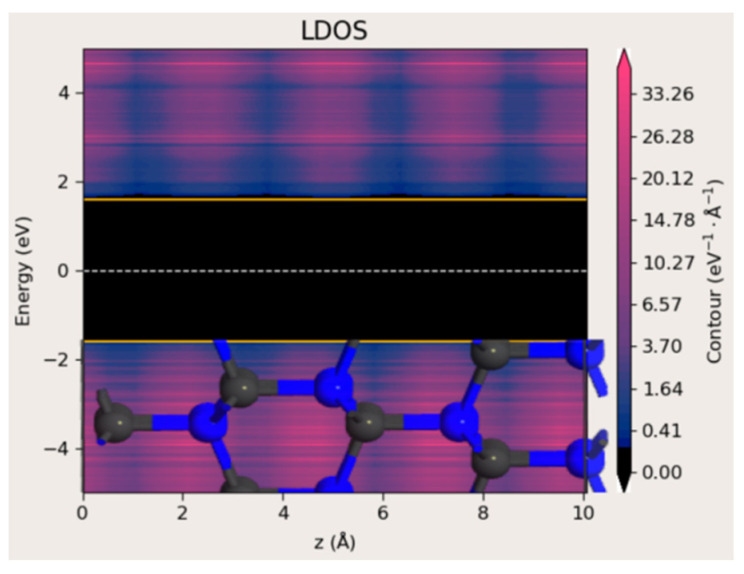
A model of a 4H-SiC crystal and LDOS as a function of position along the c-axis direction. The structure contains 200 atoms. The yellow lines indicate the energy levels at which the density of states (DOS) drops to zero, corresponding to the edges of the conduction and valence bands in the semiconducting region.

As a reference, an ideal 4H-SiC structure was first simulated to verify the accuracy of the band gap calculation. A supercell consisting of a 5 × 5 × 1 unit cell (200 atoms) was constructed. The space-resolved density of states (local DOS and LDOS) along the c-axis direction is shown in Figure 9. The calculated band gap of approximately 3.2 eV is in good agreement with reported experimental values, confirming the validity of the adopted computational approach.

**Figure 10:**
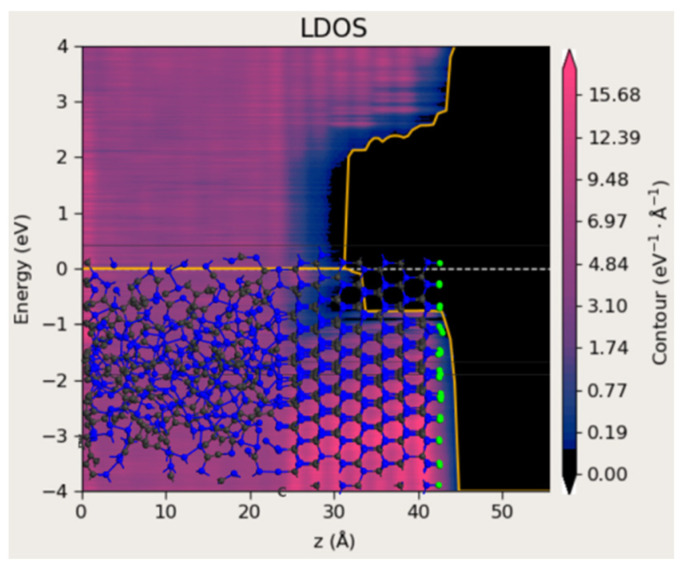
A model of the 4H-SiC and amorphous SiC interface followed by the calculated DOS as a function of position. The structure contains 927 atoms. The yellow lines indicate the spatially dependent energy levels at which the local density of states (LDOS) drops to zero, corresponding to the conduction and valence band edges across the semiconducting region and at the interface.

To visualize the effect of laser-induced structural modification, an interface between crystalline 4H-SiC and amorphous SiC (a-SiC) was constructed, as presented in Figure 10. The free surface of the 4H-SiC region was passivated with hydrogen atoms, and the atomic positions at the interface were optimized using the force field method implemented in QuantumATK. The results show that the a-SiC region exhibits no band gap, which can be attributed to the presence of a high density of dangling bonds in the amorphous phase.

**Figure 11:**
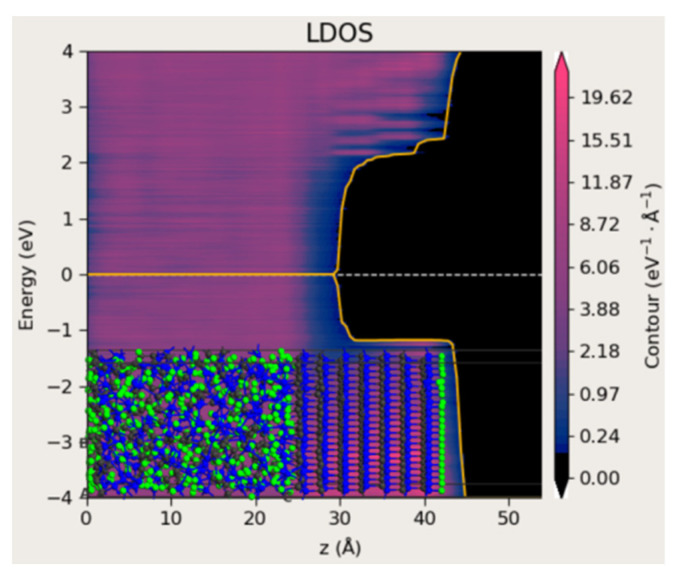
A model of 4H-SiC and amorphous SiC interface with passivated dangling bonds with LDOS. The structure contains 1340 atoms. The dangling bonds of Si and C atoms inside the amorphous structure are passivated with hydrogen atoms (green). The yellow lines indicate the spatially dependent energy levels at which the local density of states (LDOS) drops to zero.

To further analyze the origin of the missing band gap, the dangling bonds of Si and C atoms in the amorphous region were explicitly passivated with hydrogen atoms (Figure 11). Surprisingly, the calculated LDOS still revealed no band gap. This suggests that the lack of an energy gap should not be solely attributed to unpassivated dangling bonds, but rather to an intrinsic property of the amorphous SiC structure formed under rapid thermal non-equilibrium conditions.

The computational results indicate that the rapid heating and instantaneous cooling associated with nanosecond UV laser irradiation lead to the formation of amorphous SiC at the substrate surface. This amorphous layer exhibits metallic-like behavior with no energy band gap.

Consequently, the presence of this interfacial amorphous phase explains the experimentally observed transition from rectifying to ohmic contact characteristics: the barrier height at the adhesive–semiconductor interface is effectively eliminated.

## 4. Conclusions

This study has demonstrated that surface modification of silicon carbide using a pulsed ultraviolet laser with a wavelength of 355 nm enables the fabrication of low-resistivity ohmic contacts on 4H-SiC substrates. The proposed approach significantly simplifies the contact formation process by eliminating the need for conventional steps such as metal deposition, photolithography, etching, and high-temperature annealing. The experimental results confirmed that even a limited number of laser operations substantially improves the electrical properties of the contact interface, while higher repetition counts lead to nearly ideal ohmic characteristics.

Raman spectroscopy revealed that the laser-induced rapid heating and cooling of the SiC surface results in the formation of a thin amorphous SiC layer with no band gap. This assumption has been further confirmed by atomic simulations. The amorphous interfacial region is likely responsible for the absence of a Schottky barrier and the observed linear I–V characteristics.

Future work will focus on investigating the effect of laser power and pulse parameters on the electrical performance of the fabricated contacts. Additionally, optimization of the scanning trajectory is planned in order to achieve greater homogeneity of the modified contact area, which may further improve reproducibility and reliability of the proposed technique. The long-term stability of the adhesive contacts will also be a key focus of future investigations, aiming to support their suitability for application-oriented solutions.

## Figures and Tables

**Table 1:** List of experimental test substrates.

Sample	Number of Laser Scans	Laser Beam Parameters
S03	3	P = 3 W λ = 355 nm f = 20 kHz v_beam_ = 1 m/s
S10	10	
S30	30	
SK1	100	
REF	0	

## Data Availability

The original contributions presented in the study are included in the article, further inquiries can be directed to the corresponding author.
